# Effectiveness and Safety of Newer Antidiabetic Medications for Ramadan Fasting Diabetic Patients

**DOI:** 10.1155/2016/6962574

**Published:** 2016-08-24

**Authors:** Ehab Mudher Mikhael

**Affiliations:** College of Pharmacy, Clinical Pharmacy Department, Baghdad University, Baghdad, Iraq

## Abstract

Hypoglycemia is the most common side effects for most glucose-lowering therapies. It constitutes a serious risk that faces diabetic patients who fast during Ramadan (the 9th month in the Islamic calendar). New glucose-lowering classes like dipeptidyl peptidase-4 (DPP-4) inhibitors, glucagon-like peptide 1 receptor agonist (GLP-1 RA), and sodium-glucose cotransporter-2 (SGLT-2) inhibitors are efficacious in controlling blood glucose level with less tendency to induce hypoglycemia and thus may constitute a good choice for diabetic patients during Ramadan. This study reviews the safety and efficacy of newer glucose-lowering therapies during Ramadan. This study was accomplished through a careful literature search about studies that assess the benefit and side effects of these new glucose-lowering therapies during Ramadan during September 2015. Vildagliptin, sitagliptin, liraglutide, exenatide, and dapagliflozin were the only studied glucose-lowering therapies. All of the studied newer glucose-lowering therapies except dapagliflozin were associated with reduced risk to induce hypoglycemia. Gastrointestinal upset was common with the usage of liraglutide while increased thirst sensation was common with dapagliflozin. In conclusion DPP-4 inhibitors such as vildagliptin and sitagliptin may form a suitable glucose-lowering therapy option for Ramadan fasting patients.

## 1. Introduction

Fasting during Ramadan, the 9th month in the Islamic calendar, is not mandatory for patients with diabetes mellitus (DM), but many insist on fasting. This can create many health problems, especially if the fast is prolonged [1]. Glucose-lowering therapies are cornerstone for treating all type 2 DM patients to ensure tight glycemic control to prevent acute complications like hyperosmolar nonketotic coma and chronic complications such as the micro- and macrovascular complications. Hypoglycemia is the most serious and fatal complication for fasting and for many treatment options for diabetes, such as insulin and some of the oral glucose-lowering therapies, including sulfonylurea (SU) and meglitinides [2, 3]. In the last decade new classes of glucose-lowering therapies associated with reduced risk of inducing hypoglycemia have been introduced. These include incretin mimetics, such as dipeptidyl peptidase-4 (DPP-4) inhibitors, glucagon-like peptide-1 receptor agonist (GLP-1 RA), and the sodium-glucose cotransporter-2 (SGLT-2) inhibitors [4, 5]. There have been few review studies of the use of these new glucose-lowering therapies during Ramadan. Most focus only on one class of glucose-lowering therapies [6, 7]. One review discussed the benefits and drawbacks for many classes of newer glucose-lowering therapies but did not include information about SGLT-2 inhibitors. Furthermore, that study did not provide a conclusion on which medication is the best to be used during Ramadan by patients with type 2 DM [8]. This study reviews the safety and efficacy of newer glucose-lowering therapies in order to identify those that are most suitable for patients with DM during the fasting month of Ramadan.

## 2. Methods

This study was accomplished during September 2015 through a careful literature search using (PubMed, PubMed Central, and Google Scholar) for studies from 2005 to 2015 with the one or more of following keywords in English language: diabetes DPP-4 inhibitor (alogliptin, linagliptin, saxagliptin, sitagliptin, and vildagliptin), GLP-1 RA (exenatide, liraglutide, albiglutide, and lixisenatide), and SGLT-2 inhibitors (canagliflozin, dapagliflozin, ipragliflozin, and empagliflozin), in combination with the essential keyword (Ramadan). EMBASE was not searched because of funding limitations. All study types (prospective observational, randomized blinded clinical trials and randomized open-label trials) that examined the efficacy and side effects of these classes of glucose-lowering therapy on patients with type 2 DM during the fasting month of Ramadan were included. Reviews were excluded. Information from these studies were summarized in relation to study design, duration of study, number of participating patients, medications used, assessment criteria for medication safety and effectiveness, and final conclusions.

## 3. Results

A total of 16 studies were included as shown in Table 1. Full text was obtained in nine studies, abstract in four studies, and posters in three studies. Eight studies were randomized clinical trials (RCT) and eight were prospective observational studies. Information about each class of glucose-lowering therapies was summarized according to the medication used in each class and whether this medication was studied as monotherapy or as add-on therapy to other glucose-lowering therapies.

### 3.1. Dipeptidyl Peptidase-4 Inhibitors

The dipeptidyl peptidase-4 (DPP-4) inhibitors are a new class of oral glucose-lowering therapies for type 2 DM treatment. They act by inhibiting the breakdown of GLP-1, increasing its systemic concentration which leads to a significant increase in endogenous insulin secretion and a decrease in glucagon secretion. They have a glucose dependent mechanism of action, leading to a lower incidence of hypoglycemia. In clinical practice DPP-4 inhibitors are associated with 0.6–0.7% reductions in glycosylated hemoglobin (HbA_1c_) without causing weight gain [9, 10]. The first DPP-4 inhibitor, sitagliptin, was approved 10 years ago by United States government's Food and Drug Administration (FDA). Since then many DPP-4 inhibitors such as vildagliptin, saxagliptin, linagliptin, and alogliptin have been approved and are available in the pharmaceutical market [11]. Vildagliptin and sitagliptin are the most frequently studied DPP-4 inhibitors for control of type 2 DM during Ramadan. Unfortunately vildagliptin is not available in USA.

#### 3.1.1. Vildagliptin

*(1) Vildagliptin as Monotherapy*. There are only few studies that assess the benefits of vildagliptin as monotherapy for type 2 DM patients maybe because of its unlicensed use as monotherapy. In nonfasting patients vildagliptin has nearly similar effectiveness to SU in lowering HbA_1c_ but with less risk to induce hypoglycemia [12]. In Ramadan-fasting type 2 DM patients, vildagliptin usage was assessed in three studies only. One of these studies was a small scale, multicenter, open-label, 4-week, observational study [13] for 97 Indian patients with type 2 DM who were fasting during Ramadan. Patients were divided into two groups at which in one group 55 patients were given vildagliptin and in the other 42 patients were given SU. The incidence of hypoglycemia (defined as blood glucose level less than 70 mg/dL; 3.9 mmol/L), which was assessed depending on patient symptoms and confirmed by measuring blood glucose level, was lower in the vildagliptin group than in SU group (0% versus 4.8%; *p* = 0.104). HbA_1c_ was decreased in vildagliptin group while there was a slight increase in SU group (−0.43% versus 0.01%; *p* < 0.05). More patients in the vildagliptin group achieved HbA_1c_ < 7.0% than in the SU treated group (16.4% versus 4.8%; *p* = 0.055). Additionally, there was a significant difference in weight loss. Patients in the vildagliptin group lost an average of 1.2 kg while those in SU group lost an average of 0.03 kg (*p* < 0.001). Although vildagliptin was shown to be safer than SU in this study, this superior safety was lacking statistical significance, perhaps due to the small sample size. In another large, multiregional, observational study [14] that was conducted in Asia and the Middle East, 1315 type 2 diabetic Muslim patients were divided into two groups where 684 patients had received treatment with vildagliptin and 631 patients received SU (glibenclamide, glimepiride, gliclazide, or glipizide) as monotherapy or as add-on to metformin. Vildagliptin was significantly more effective in reducing HbA_1c_ than SU (−0.24% versus 0.02%; *p* < 0.05). Also, vildagliptin was associated with significantly fewer episodes of hypoglycemic events (defined as patient reported symptoms and/or blood glucose level less than 70 mg/dL; 3.9 mmol/L) in comparison with the SU therapy (5.4% versus 19.8%; *p* < 0.05). This large study confirmed that vildagliptin had significantly higher effectiveness and safety when compared to SU. These two studies showed that the risk of hypoglycemia in patients using vildagliptin is around one-third to that in patients using SU.

In summary, the use of vildagliptin 50 mg twice daily as monotherapy for Ramadan fasting patients is more effective than SU to control blood glucose level (through HbA_1c_ reduction) and body weight. It is also safer than SU by its less risk to induce hypoglycemia.

*(2) Vildagliptin as Add-On Therapy*. There are many studies examining vildagliptin as add-on therapy for both fasting and nonfasting patients. In nonfasting patients it was found that vildagliptin when used as add-on therapy to metformin has comparable efficacy to different SU (glimepiride and gliclazide) but with less risk to induce hypoglycemia [15, 16]. In Ramadan fasting patients, three studies assess vildagliptin in Ramadan fasting type 2 DM patients. The 1st study in this regard was a small scale study [17] that was conducted in London and included 52 patients with type 2 DM who were already using metformin (2 g/day); these patients were randomized equally into two groups where half of them were given vildagliptin 50 mg daily and the other half were given gliclazide 160 mg twice daily in addition to their primary therapy. Hypoglycemic events (defined as blood glucose <63 mg/dL; 3.5 mmol/L with or without symptoms) significantly occurred less frequently for patients in the vildagliptin group than in the gliclazide group (7.7% versus 61.5%; *p* ≤ 0.001). The effect of both gliclazide and vildagliptin was similar on HbA_1c_ and body weight. The lack of significant difference in the effectiveness between vildagliptin and gliclazide in this study may be due to short period (24 days) of follow-up besides the small sample size which further compromises statistical significance. Another small prospective, 16-week, multicenter study [18] was conducted in UK for patients who were using either vildagliptin 50 mg twice a day (30 patients) or gliclazide (41 patients) as add-on therapy to metformin during Ramadan fasting. There were no hypoglycemic events (defined as blood glucose level less than 70 mg/dL; 3.9 mmol/L) for patients in the vildagliptin group while 44.4% of the patients in the SU group suffered from hypoglycemic events. There was significantly greater HbA_1c_ reduction for those taking vildagliptin; however this higher effectiveness may be attributed to higher adherence rate by patients using vildagliptin than for those using gliclazide [19]. On the other hand the high missing rate among patients using gliclazide is a confirmation to the finding of this study in that vildagliptin despite regular usage resulted in fewer hypoglycemic events.

In 2014 a randomized, open-label, clinical trial [20] was done on 69 patients who were on a combination of metformin and SU (glimepiride or gliclazide). Patients were divided into two groups: a control group in which patients were maintained on their usual treatment regimen with dose adjustment for the fasting period and a study group at which patients were switched from SU to vildagliptin 50 mg twice daily in combination with metformin. There was no difference in the effect of vildagliptin and SU on the calculated change in HbA_1c_. The incidence of hypoglycemia, which was confirmed by measuring blood glucose level, during Ramadan was higher in the SU group (26 episodes versus 19 episodes; *p* = 0.334). The number of patients who had medication noncompliance because of fasting discomfort was higher in the SU than in the vildagliptin group.

All the previously discussed small scale studies [17, 18, 20] concluded that vildagliptin as add-on therapy to metformin was at least as effective as SU with a lower risk to induce hypoglycemia for type 2 DM patients during Ramadan.

Another large study was a prospective, observational, 14-week study design [21] for 198 stable patients on dual oral therapy for ≥2 months and with HbA_1c_ ≤ 8.0%. 83 patients were in the metformin-sulfonylurea/glinide (IS) cohort and 115 patients were in the metformin-vildagliptin cohort. Hypoglycemic episodes (defined as blood glucose ≤ 70; 3.9 mmol/L) were confirmed in 30.8% of the IS cohort and 23.5% in the vildagliptin cohort (*p* > 0.05), while severe hypoglycemia and/or unscheduled medical visit due to hypoglycemia occurred in 10.4% of the IS cohort and in 2.6% of vildagliptin cohort (*p* = 0.0029). Glycemic control remained stable in both cohorts. Compliance with fasting was higher, as well as adherence to drug therapy in vildagliptin cohort, with ≥5 missed doses for 15.4% of IS, compared to 8.5% only in patients using vildagliptin. In this study the nonsignificant difference in the total hypoglycemic episodes between vildagliptin and SU/glinide which was different from the finding of previous studies at which vildagliptin was less likely to induce hypoglycemia than SU, maybe due to the usage of glinide in some patients but unfortunately their number was vague which keeps the final conclusion about the hypoglycemic risk of glinide in comparison with vildagliptin unknown.

Another study, VIRTUE study [22] was a multicenter, prospective, 16-week observational study that enrolled 244 Pakistani patients with type 2 DM. All included patients were already treated with vildagliptin (*n* = 121) or SU (*n* = 121; 67% on glimepiride, 14% on gliclazide, 18% on glibenclamide, and 1% on glipizide) as add-on to metformin or as monotherapy for at least 4 weeks. Patients in the vildagliptin group experienced at least one episode of hypoglycemia (defined as blood glucose measurement ≤ 70.2 mg/dL; 3.9 mmol/L) less frequently than patients in the SU group (5.8% versus 14.2%; *p* < 0.033). The reduction in HbA_1c_ was greater with vildagliptin than in SU (−0.3% versus −0.1%; *p* < 0.054). A reduction of 0.3 kg in body weight was seen with vildagliptin treatment versus 0.2 kg weight gain in the SU group. Overall adverse events (hypoglycemia, nausea, vomiting, abdominal pain, and abdominal discomfort) were less frequently reported in vildagliptin cohort than in the SU group (15.7% versus 17.4%; *p* = 0.729). Hypoglycemic events were significantly less common in vildagliptin than in SU group (5% versus 13.2%; *p* = 0.024). GIT side effects including abdominal pain, nausea, and vomiting are more common in vildagliptin than in SU group (10.8% versus 2.5%; *p* < 0.05); these side effects may be symptoms for acute pancreatitis which is a rare but serious side effect of vildagliptin therapy [23].

However, large, multiregional, randomized studies are required to confirm the safety of vildagliptin on GIT system and/or pancreatitis when used as add-on therapy to metformin for Ramadan fasting patients since this study was just an observational study on a limited number of Pakistani patients only.

More recently a STEADFAST study [24] was a multiregional, randomized, double-blinded study for 557 patients with type 2 DM who were previously treated with metformin and any SU were randomized to receive vildagliptin 50 mg twice daily or gliclazide plus metformin. The percent of patients reporting confirmed hypoglycemia (blood glucose less than <70.2 mg/dL; 3.9 mmol/L) were lower with vildagliptin than the gliclazide (3% versus 7.0%; *p* = 0.039). There was a nonsignificant difference in effectiveness of vildagliptin versus gliclazide according to the adjusted mean change in HbA_1c_ (*p* = 0.165). Also there was a nonsignificant difference between vildagliptin and gliclazide on body weight change (*p* = 0.987). Overall safety (measured by adverse effects on all body organs) was similar between the treatments. The randomized design of this study and its large sample size lead to a less biased conclusions and a thus form a strong evidence for the lower risk of hypoglycemia with comparable safety of vildagliptin when compared to gliclazide. This finding occurs in contrast to the findings of VIRTUE study which assume that GIT upset is higher by vildagliptin than in SU (the majority of patients were on glimepiride) while in STEADFAST study vildagliptin has similar incidence of GIT side effects to gliclazide and since the incidence of GIT side effects is higher by glimepiride than gliclazide [25]; then it can be concluded that vildagliptin has at least comparable safety to SU.

In summary, the usage of vildagliptin in fasting patients as add-on therapy to metformin has comparable safety and effectiveness to SU but with a lower tendency to induce hypoglycemia.

#### 3.1.2. Sitagliptin

*(1) Sitagliptin as Add-On Therapy*. There is a complete absence for studies that evaluate the effect of sitagliptin as monotherapy for patients with type 2 DM during Ramadan but there are many studies on the usage of sitagliptin as add-on therapy to other glucose-lowering therapies, specifically metformin. In a pilot prospective study [26] involving 15 patients, a combination of sitagliptin with metformin was safe (not associated with hypoglycemic events) for Ramadan fasting patients with type 2 DM; however, the small sample size and the funding of this study by a pharmaceutical company that manufactures sitagliptin make it difficult to draw an accurate conclusion regarding the benefits of using sitagliptin during Ramadan.

Sitagliptin had been also studied in large, randomized, open-label multinational study [27] that was done on 1021 type 2 DM patients who intended to fast during Ramadan and were already treated with stable doses of SU (35% glibenclamide, 35% glimepiride, or 30% gliclazide) and metformin for at least 3 months before screening. Patients were randomized to either receive sitagliptin 100 mg/day plus metformin (*n* = 507) or remain on their prestudy treatment (*n* = 514). There was a nonsignificant difference in the incidence of symptomatic hypoglycemia (based on patient reported symptoms and confirmed by blood glucose level less than 70 mg/dL; 3.9 mmol/L) for patient in the gliclazide group when compared with those in the sitagliptin (6.6% versus 6.7%; *p* > 0.05) group, while a significant difference in the incidence of hypoglycemia occurs between patients using sitagliptin and those using glibenclamide and glimepiride. Furthermore, sitagliptin appeared to induce side effects to a lesser extent than SU (3 versus 9 patients, resp.); all the side effects in sitagliptin group were not serious and include constipation, vomiting, or hyperglycemia, while three patients in SU group developed serious problems like ischemic stroke, acute pancreatitis, and urinary tract infection. In this study although there was a nonsignificant difference in the incidence of hypoglycemia between gliclazide and sitagliptin, gliclazide was associated with lower risk of hypoglycemia when compared to sitagliptin; unfortunately authors in that study did not classify patients who are using gliclazide according to the used dosage form (sustained and immediate release), so it is difficult to conclude that whether this lower incidence of hypoglycemia by gliclazide when compared to sitagliptin is related to specific dosage form of gliclazide or due to gliclazide itself.

Another multicenter, randomized study [28] is involving 848 Ramadan fasting patients with type 2 DM, who were already treated with a stable dose of SU (65% glimepiride, 22% glibenclamide, and 13% gliclazide) with or without metformin (86% and 14%, resp.) for ≥3 months and had HbA_1c_ ≤ 10%. Patients were divided into two groups: 421 patients were switched from SU to sitagliptin 100 mg once daily and 427 patients remained on SU. The proportion of patients who recorded ≥1 symptomatic hypoglycemic event during Ramadan was lower for patients in sitagliptin than in SU group (3.8% versus 7.3%; *p* = 0.028). The incidence of symptomatic hypoglycemia was the lowest in patients using gliclazide (1.8%), then sitagliptin (3.8%), then glibenclamide (5.2%), and finally glimepiride (9.1%). The proportion of patients experiencing adverse effects other than hypoglycemia was 10.0% versus 7% in the sitagliptin and SU group, respectively. The major limitation for this study was the absence of medication efficacy assessment through measuring glycemic control and body weight. The assumption of this study in that sitagliptin is safer than SU in regard to hypoglycemia was not accurate since incidence of hypoglycemia is lower in gliclazide than in sitagliptin. The finding of this study may provide confirmatory evidence to the finding of Al Sifri et al. [27] which found a less likely risk of hypoglycemia by gliclazide when compared to sitagliptin. Furthermore, failure to assess the effect of sitagliptin versus SU on glycemic control and weight for fasting patients in these studies can be considered as main limitation in drawing a reliable conclusion regarding the usage of sitagliptin during Ramadan [26–28].

In summary, the studies that evaluate the usage of sitagliptin for patients with diabetes during Ramadan found that it is associated with reduced risk of hypoglycemia when used as add-on therapy to metformin compared to two SUs (glimepiride and glibenclamide) and a slightly higher risk of hypoglycemia than gliclazide; this difference may be attributed to the higher degree of selectivity in pancreatic receptor stimulation by gliclazide [29] while in nonfasting patients the risk of hypoglycemia was lower in sitagliptin than SU [30].

Other rare side effects including gastrointestinal (GIT) side effects (vomiting, constipation, and abdominal pain) and central nervous system (CNS) side effects (headache, dizziness, and decreased concentration) appeared to occur at higher percent in sitagliptin than in SU treated patients. One of the major limitations in all of these studies about the usage of sitagliptin during Ramadan is that they did not focus on sitagliptin effect to control blood glucose level, so further studies are needed in this regard to find out whether sitagliptin benefit is limited to less risk of hypoglycemia or extends beyond that to include a better glycemic control than SU for patients with type 2 DM during Ramadan.

#### 3.1.3. Other DPP-4 Inhibitors

Till the time of collecting the data for this review there are no any study (2005–2015) that had been evaluating the effect and side effects of using DPP-4 other than vildagliptin and sitagliptin like linagliptin, saxagliptin, and alogliptin during Ramadan. It seems that the differences are negligible regarding efficacy and incidence of hypoglycemia among all DPP-4 inhibitors [11]. So it may be reasonable for researchers to investigate the benefits of other new DPP-4 inhibitors in countries at which vildagliptin and sitagliptin are not available.

### 3.2. Glucagon-Like Peptide-1 Receptor Agonists

Glucagon-like peptide-1 receptor agonists (GLP-1 RA) act by binding to and activating the GLP-1 receptor, resulting in glucose dependent increase in insulin secretion and decrease secretion of glucagon, so they are effective in decreasing blood glucose levels associated with reduced risk of hypoglycemia. They also act to delay gastric emptying and increase satiety; thereby they are effective in reducing body weight for diabetic patients. The approved agents in this class include exenatide, liraglutide, albiglutide, and lixisenatide. All of these agents are administered by subcutaneous injections with mainly GIT side effects such as nausea, vomiting, and diarrhea in addition to injection-site reaction [31, 32]. Exenatide and liraglutide are the most frequently studied GLP-1 RAs for patients with diabetes during Ramadan.

#### 3.2.1. Exenatide as Add-On Therapy

Although there are many studies that examine the effect of exenatide as add-on therapy in nonfasting patients [33, 34], which showed that exenatide was effective to lower HbA_1c_ with less hypoglycemic risk, only few studies examined exenatide usage for Ramadan fasting patients. One pilot study [35] for 34 patients with type 2 DM who are using different pharmacological treatment (insulin and oral glucose-lowering therapies) and are wishing to fast during Ramadan was observed. Two patients were using exenatide prior to Ramadan. At the end of Ramadan, it was found that neither of the two patients had experienced a hypoglycemic event (defined as blood glucose less than 70 mg/dL; 3.9 mmol/L) even without exenatide dose adjustment. However, it is so difficult to ascertain this result because of the limited sample size. In another study, exenatide when used as add-on therapy to metformin [36] was associated with reduced risk of hypoglycemia when compared to a combination of metformin and gliclazide for patients with type 2 DM who fast during Ramadan. One limitation of this study is inability of the author to retrieve the full article.

In above 2 studies regular exenatide was assessed while the usage of sustained release exenatide during Ramadan was not assessed.

In summary, regular exenatide is not associated with hypoglycemia when used for type 2 DM patients during Ramadan; however it is difficult to recommended the use of exenatide for fasting patients until further studies performed because the current studies are small scaled studies, with major focus on hypoglycemic side effects without focusing on the efficacy of exenatide to control blood glucose level during Ramadan.

#### 3.2.2. Liraglutide as Add-On Therapy

Liraglutide was shown to have comparable efficacy to SU in lowering HbA_1c_ but with less risk of hypoglycemia when used in nonfasting patients [37, 38].

During Ramadan many studies assess the benefits and drawbacks for liraglutide usage; one of the earliest studies in this regard was the Treat 4 Ramadan trial, which was a randomized, controlled clinical trial [39] comparing liraglutide to SU (gliclazide 88%, glimepiride 10%, or glibenclamide 2%) as add-on therapy to metformin in 99 adult patients with type 2 DM in UK. After 12 weeks, patients in the liraglutide group and not those in the SU group had a reduction in HbA_1c_ (−0.3% versus 0.02%; *p* = 0.06). Liraglutide resulted in greater and significant reductions in both weight and diastolic blood pressure (BP) for patients with DM than SU. Self-recorded episodes of hypoglycemia (blood glucose ≤ 70.2 mg/dL; 3.9 mmol/L) were significantly lower with liraglutide (*p* < 0.0001). The major limitation in this study was the reliability of the method that was used to calculate hypoglycemia (patient self-record method).

LIRA- Ramadan study was longer and larger than the previous Treat 4 trial. LIRA study was an open-label, multinational randomized clinical trial [40] involving 343 people (172 on liraglutide and 171 on SU) for a 33-week duration. This study included type 2 DM patients with an intent to fast during Ramadan, with HbA_1c_ 7–10%, and being treated with a combination of metformin and SU (at maximum tolerated dose). Study participants were randomized to either switch from SU to liraglutide 1.8 mg once daily or continue pretrial SU. Patients in liraglutide group were more likely to achieve HbA_1c_ target of <7% with no confirmed hypoglycemic events (defined as blood glucose less than 70 mg/dL; 3.9 mmol/L) compared with SU (53.9% versus 23.5%; *p* < 0.0001). Moreover, people treated with liraglutide experienced significantly greater weight loss (*p* < 0.0001) and greater improvements in HbA_1c_ (−1.24% versus −0.65%; *p* < 0.0001) than those treated with SU. The incidence of patients experiencing adverse events (AE) during Ramadan was similar in the liraglutide and SU groups (23.7% versus 20.9%; *p* > 0.05); meanwhile gastrointestinal side effects (nausea, diarrhea, vomiting, abdominal pain, and abdominal distension) occurred more commonly with liraglutide treatment (10.5% versus 3.7%). The results of this study were more reliable and add a confirmation to the findings of Treat 4 trial because of its randomized design with larger sample size at which in both studies liraglutide showed a significantly better efficacy than SU with a lower incidence of hypoglycemia.

In summary, liraglutide usage during Ramadan for patients with type 2 DM may be reasonable because it is associated with better glycemic control, improved body weight, and less hypoglycemic episodes when compared with SU; however, it should be used with caution because of its GIT side effects, which may negatively affect a fasting patient, since GIT problems may occur more frequently during Ramadan [41].

#### 3.2.3. Other GLP-1 RA

Studies for other GLP-1 RA like albiglutide and lixisenatide during Ramadan were lacking which may be attributed to their recent approval by FDA and it may be possible to find such trials in the near future.

### 3.3. Sodium-Glucose Cotransporter-2 Inhibitors

Sodium-glucose cotransporter-2 (SGLT-2) inhibitors are the most recent class of oral glucose-lowering therapies that are used for treating patient with type 2 DM. Medications of this class include dapagliflozin, canagliflozin, ipragliflozin, and empagliflozin. These drugs act to lower blood glucose level by decreasing renal glucose threshold through their effect to induce a competitive inhibition on the SGLT-2 in the kidney which is responsible for reabsorption of 90% of filtered glucose by the kidneys and thus block the reabsorption of glucose. The risk of inducing hypoglycemia is low with SGLT-2 inhibitors because of their insulin-independent action and hence forms an attractive class for managing patients with type 2 DM during Ramadan. However, caution is recommended while using these medications because of their ability to cause dehydration, especially in the setting of absence of fluid intake, which occurs during fasting hours of Ramadan [42, 43]. Recently FDA added many warning on such class of medication because of their risk to induce ketoacidosis and increase risk of foot and leg amputation, serious urinary tract infections, acute renal failure, and osteoporosis [44].

#### 3.3.1. Dapagliflozin as Add-On Therapy

There are some studies that assess dapagliflozin as add-on therapy in nonfasting patients; in all of these studies dapagliflozin was shown to have comparable efficacy to SU with lower hypoglycemic risk [45].

To date, there is only one study that evaluates dapagliflozin during Ramadan which was a 12-week, randomized, open-label study [46] for 110 patients with type 2 DM who were already using metformin and SU. Patients were divided into two groups: in the 1st group 58 patients were switched from SU to dapagliflozin 10 mg once daily while in the 2nd group 52 patients were remained on their pretrial treatment. Dehydration was defined as a loss of 1.8% of body weight/13 hours of fasting daily. Dehydration was further assessed by using urine and blood tests, with physical examination and a specific set of questions to the patients about their medical history while using this medication. There was no significant difference in the incidence of dehydration between dapagliflozin and SU (73.1% versus 81.6%; *p* = 0.258); this may be because already most of Ramadan fasting persons without regard to their disease or medication status suffer from dehydration due to long period (12–22 hour) of abstinence from foods and water during Ramadan [47].

There were significantly more patients in the dapagliflozin group (43.1% versus 23.1%; *p* = 0.026) than in SU group complained from thirst sensation. Additionally, there was a significantly higher mean for haematocrit level (*p* = 0.009), urine osmolarity (*p* = 0.001), and blood ketone (*p* = 0.002) in dapagliflozin group; however, there was a lack of information regarding the development of ketoacidosis in participated patients of this study. Furthermore, there was a significantly lower mean of urinary sodium (*p* < 0.005) in dapagliflozin group when compared to SU group; however authors of that study postulated that dapagliflozin does not pose a higher risk of dehydration during Ramadan. Assessment of dapagliflozin effectiveness to reduce HbA_1c_ and its risk to induce hypoglycemia was the major limitation in this study which may be because the main aim of this study was to assess the safety of dapagliflozin on excessive water excretion.

In summary, there are a limited number of trials regarding dapagliflozin and other SGLT-2 inhibitors in type 2 DM patients during Ramadan. The available data showed that although dapagliflozin is not associated with increased risk of dehydration, it increases thirst sensation for patients with type 2 DM during Ramadan, which may negatively affect patient compliance to continue dapagliflozin usage during Ramadan. So it is recommended not to use SGLT-2 inhibitors for fasting patients with type 2 DM until performing further studies that compare the effect of dapagliflozin or other SGLT-2 inhibitors with other oral antidiabetic medications on controlling blood glucose level, their risk of inducing hypoglycemia, and patient compliance.

*Limitations of the Current Study*. There are many limitations in this review like the usage of free search engines for literature search and the inability to fully retrieve some articles.

## 4. Conclusion

Although many glucose-lowering therapies with noninsulin dependent mechanisms of action have been approved recently, only few of them (vildagliptin, sitagliptin, exenatide, liraglutide, and dapagliflozin) have been studied during Ramadan. The hypoglycemic risk was assessed for all of the above medications except dapagliflozin; nearly all of the assessed medications were associated with reduced risk of hypoglycemia when compared with SU when used during Ramadan. DPP-4 inhibitors such as vildagliptin and sitagliptin may form a suitable glucose-lowering therapy option for Ramadan fasting patients, since they are unlike liraglutide less likely to cause GIT upset, and unlike SGLT-2 inhibitors are not associated with increased thirst sensation. Effectiveness of sitagliptin to control blood glucose level for Ramadan fasting patients was not assessed in any study in contrast to vildagliptin which was shown to be effective to control blood glucose level and body weight when used as monotherapy or even as add-on therapy to metformin; accordingly vildagliptin seems to be the most suitable glucose-lowering therapy choice for diabetic patients who are wishing to fast during Ramadan. Further studies on the use of other new glucose-lowering therapies during Ramadan are recommended; furthermore it is recommended to do studies that directly compare the advantages and disadvantages between these new glucose-lowering therapies.

## Figures and Tables

**Table 1:** Summary of the included studies.

Class studied	Medication	Type of study	Research conclusions
DPP-4 inhibitors	Vildagliptin as monotherapy	Small scale observational study	Vildagliptin was significantly more effective than SU to reduce HbA_1c_ and body weight; vildagliptin is associated with fewer hypoglycemic episodes than SU [12]
		large scale multiregional observational study	Vildagliptin was significantly more effective to reduce HbA_1c_ and less inducing hypoglycemia than SU [13]

DPP-4 inhibitors	Vildagliptin as add-on therapy	Randomized small scale study	Vildagliptin has similar effectiveness on HbA_1c_ and body weight to gliclazide; vildagliptin was significantly less inducing of hypoglycemia than gliclazide [14]
		Small scale, observational prospective study	Vildagliptin was not associated with hypoglycemic events while nearly half of the patients in the SU group suffered from hypoglycemic events. There was significantly greater HbA_1c_ reduction by vildagliptin than SU [15]
		Small scale, randomized open-label study	Vildagliptin was less significantly associated with hypoglycemia but with similar effectiveness on HbA_1c_ to SU [17]
		Large, prospective observational study	Vildagliptin was associated with fewer episodes of severe hypoglycemia but with similar glycemic control to SU/glinide [18]
		Large, multicenter, prospective observational study	Vildagliptin using patients suffered from hypoglycemia less frequently than those using SU. The reduction in HbA_1c_ was greater but not significantly different with vildagliptin than in SU [19]
		Multiregional, large scale, randomized double blind study	Vildagliptin had similar effectiveness to lower HbA_1c_ but with less hypoglycemic risk than gliclazide [20]

DPP-4 inhibitors	Sitagliptin as add-on therapy	Pilot prospective observational study	Sitagliptin usage was not associated with hypoglycemic attacks [22]
		Large, multinational randomized study	Sitagliptin was associated with significantly less risk of hypoglycemia than glibenclamide and glimepiride but similar risk to gliclazide [23]
		Large, multicenter, randomized study	Sitagliptin was associated with significantly less risk of hypoglycemia than glibenclamide and glimepiride but higher hypoglycemic risk than gliclazide [24]

GLP-1 RA	Exenatide as add-on therapy	Pilot observational study	No risk of hypoglycemia even if pre-Ramadan dose of exenatide is not adjusted during Ramadan [27]
		Observational study	Exenatide was associated with less risk of hypoglycemia than gliclazide [28]

GLP-1 RA	Liraglutide as add-on therapy	Small scale randomized study	Liraglutide was associated with significant reduction in body weight and nonsignificant but greater reduction in HbA_1c_ than SU Liraglutide was less significantly inducing hypoglycemia than SU [29]
		Large, open-label, multinational randomized trial	Liraglutide was less likely to produce confirmed hypoglycemic attacks compared with SU. Moreover, patients on liraglutide experienced significantly greater weight loss and had significantly greater improvements in HbA_1c_ than those on SU [30]

SGLT-2 inhibitors	Dapagliflozin as add-on therapy	Randomized open-label study	There was a nonsignificant difference in the incidence of dehydration between patients in the dapagliflozin and SU group [34]

DPP-4: dipeptidyl peptidase-4 inhibitor; GLP-1 RA: glucagon-like peptide-1 receptor agonist; SGLT-2: sodium-glucose cotransporter-2; SU: sulfonylurea; HbA_1c_: glycosylated hemoglobin.

## References

[1] Chamsi-Pasha H., Aljabri K. (2014). The diabetic patient in Ramadan. *Avicenna Journal of Medicine*.

[2] Chowdhury T. A. (2011). Severe hypoglycemia in a Muslim patient fasting during Ramadan. *Diabetic Hypoglycemia*.

[3] Ahmad J., Pathan M. F., Jaleel M. A. (2012). Diabetic emergencies including hypoglycemia during Ramadan. *Indian Journal of Endocrinology and Metabolism*.

[4] Jaleel M. A., Raza S. A., Fathima F. N., Jaleel B. N. (2011). Ramadan and diabetes: As-Saum (The fasting). *Indian Journal of Endocrinology and Metabolism*.

[5] Gude D. (2012). Red carpeting the newer antidiabetics. *Journal of Pharmacology & Pharmacotherapeutics*.

[6] Aziz K. M. A. (2015). Fasting during Ramadan: efficacy, safety, and patient acceptability of vildagliptin in diabetic patients. *Diabetes, Metabolic Syndrome and Obesity: Targets and Therapy*.

[7] Schweizer A., Halimi S., Dejager S. (2014). Experience with DPP-4 inhibitors in the management of patients with type 2 diabetes fasting during Ramadan. *Vascular Health and Risk Management*.

[8] Bajaj S. (2015). Newer glucose-lowering therapy drugs in Ramadan. *Journal of the Pakistan Medical Association*.

[9] Reid T. (2012). Choosing GLP-1 receptor agonists or DPP-4 inhibitors: weighing the clinical trial evidence. *Clinical Diabetes*.

[10] McDougall C., McKay G. A., Fisher M. (2011). Drugs for diabetes: part 5 DPP-4 inhibitors. *British Journal of Cardiology*.

[11] Dicker D. (2011). DPP-4 inhibitors: impact on glycemic control and cardiovascular risk factors. *Diabetes Care*.

[12] Foley J. E., Sreenan S. (2009). Efficacy and safety comparison between the DPP-4 inhibitor vildagliptin and the sulfonylurea gliclazide after two years of monotherapy in drug-naïve patients with type 2 diabetes. *Hormone and Metabolic Research*.

[13] Shete A., Shaikh A., Nayeem K. J. (2013). Vildagliptin vs sulfonylurea in Indian Muslim diabetes patients fasting during Ramadan. *World Journal of Diabetes*.

[14] Al-Arouj M., Hassoun A. A. K., Medlej R. (2013). The effect of vildagliptin relative to sulphonylureas in Muslim patients with type 2 diabetes fasting during Ramadan: The VIRTUE Study. *International Journal of Clinical Practice*.

[15] Ferrannini E., Fonseca V., Zinman B. (2009). Fifty-two-week efficacy and safety of vildagliptin vs. glimepiride in patients with type 2 diabetes mellitus inadequately controlled on metformin monotherapy. *Diabetes, Obesity and Metabolism*.

[16] Filozof C., Gautier J.-F. (2010). A comparison of efficacy and safety of vildagliptin and gliclazide in combination with metformin in patients with type 2 diabetes inadequately controlled with metformin alone: A 52-Week, Randomized Study. *Diabetic Medicine*.

[17] Devendra D., Gohel B., Bravis V. (2009). Vildagliptin therapy and hypoglycaemia in Muslim type 2 diabetes patients during Ramadan. *International Journal of Clinical Practice*.

[18] Hassanein M., Hanif W., Malik W. (2011). Comparison of the dipeptidyl peptidase-4 inhibitor vildagliptin and the sulphonylurea gliclazide in combination with metformin, in Muslim patients with type 2 diabetes mellitus fasting during Ramadan: results of the VECTOR study. *Current Medical Research and Opinion*.

[19] Hanif W., Malik W., Hassanein M. (2013). Treatment adherence with vildagliptin compared to sulphonylurea as add-on to metformin in Muslim patients with type 2 diabetes mellitus fasting during Ramadan. *Current Medical Research and Opinion*.

[20] Malha L. P., Taan G., Zantout M. S., Azar S. T. (2014). Glycemic effects of vildagliptin in patients with type 2 diabetes before, during and after the period of fasting in Ramadan. *Therapeutic Advances in Endocrinology and Metabolism*.

[21] Halimi S., Levy M., Huet D., Quéré S., Dejager S. (2013). Experience with vildagliptin in type 2 diabetic patients fasting during Ramadan in France: insights from the VERDI Study. *Diabetes Therapy*.

[22] Mahar S. A., Hasan M. I., Khan M. I. H. (2014). Comparison of hypoglycaemia episodes in people with type-2 diabetes fasting in Ramazan, treated with vildaglipton or sulphonylurea: results of the Pakistani cohort of the VIRTUE study. *Journal of the Pakistan Medical Association*.

[23] Girgis C. M., Champion B. L. (2011). Vildagliptin-induced acute pancreatitis. *Endocrine Practice*.

[24] Hassanein M., Abdallah K., Schweizer A. (2014). A double-blind, randomized trial, including frequent patient-physician contacts and Ramadan-focused advice, assessing vildagliptin and gliclazide in patients with type 2 diabetes fasting during Ramadan: the STEADFAST study. *Vascular Health and Risk Management*.

[25] Chipirishetti R., Bondu A. K., Adil M. S., Challa R. R. (2015). Comparative study of glimepiride and gliclazide in type 2 diabetes patients on safety, efficacy and tolerability. *Journal of Drug Delivery and Therapeutics*.

[26] Al-Sayed M. Y., Joudeh M. Religious fasting in patients with Type 2 diabetes mellitus using sitagliptin/metformin-based therapy.

[27] Al Sifri S., Basiounny A., Echtay A. (2011). The incidence of hypoglycaemia in Muslim patients with type 2 diabetes treated with sitagliptin or a sulphonylurea during Ramadan: a randomised trial. *International Journal of Clinical Practice*.

[28] Aravind S. R., Ismail S. B., Balamurugan R. (2012). Hypoglycemia in patients with type 2 diabetes from India and Malaysia treated with sitagliptin or a sulfonylurea during Ramadan: A Randomized, Pragmatic Study. *Current Medical Research and Opinion*.

[29] Abdelmoneim A. S., Hasenbank S. E., Seubert J. M., Brocks D. R., Light P. E., Simpson S. H. (2012). Variations in tissue selectivity amongst insulin secretagogues: a systematic review. *Diabetes, Obesity and Metabolism*.

[30] Arechavaleta R., Seck T., Chen Y. (2011). Efficacy and safety of treatment with sitagliptin or glimepiride in patients with type 2 diabetes inadequately controlled on metformin monotherapy: a randomized, double-blind, non-inferiority trial. *Diabetes, Obesity and Metabolism*.

[31] Su-Ynn C. (2011). How do incretin-based therapies fit into the treatment algorithm?. *The Singapore Family Physician*.

[32] Trujillo J. M., Nuffer W., Ellis S. L. (2015). GLP-1 receptor agonists: a review of head-to-head clinical studies. *Therapeutic Advances in Endocrinology and Metabolism*.

[33] DeFronzo R. A., Ratner R. E., Han J., Kim D. D., Fineman M. S., Baron A. D. (2005). Effects of exenatide (exendin-4) on glycemic control and weight over 30 weeks in metformin-treated patients with type 2. *Diabetes Care*.

[34] Kendall D. M., Riddle M. C., Rosenstock J. (2005). Effects of exenatide (exendin-4) on glycemic control over 30 weeks in patients with type 2 diabetes treated with metformin and a sulfonylurea. *Diabetes Care*.

[35] Lessan N., Hasan H., Barakat M. T. (2012). Ramadan fasting: a study of changes in glucose profiles among patients with diabetes using continuous glucose monitoring. *Diabetes Care*.

[36] Bravis V., Hui E., Salih S. (2010). A comparative analysis of exenatide and gliclazide during the month of Ramadan. *Diabetic Medicine*.

[37] Nauck M., Frid A., Hermansen K. (2013). Long-term efficacy and safety comparison of liraglutide, glimepiride and placebo, all in combination with metformin in type 2 diabetes: 2-year results from the LEAD-2 study. *Diabetes, Obesity and Metabolism*.

[38] Nauck M., Frid A., Hermansen K. (2009). Efficacy and safety comparison of liraglutide, glimepiride, and placebo, all in combination with metformin, in type 2 diabetes. *Diabetes Care*.

[39] Brady E. M., Davies M. J., Gray L. J. (2014). A randomized controlled trial comparing the GLP-1 receptor agonist liraglutide to a sulphonylurea as add on to metformin in patients with established type 2 diabetes during Ramadan: the Treat 4 Ramadan Trial. *Diabetes, Obesity and Metabolism*.

[40] Azar S., Echtay A., Mohamad W. Efficacy and safety of liraglutide versus sulfonylurea both in combination with metformin during Ramadan in subjects with type 2 diabetes (LIRA-Ramadan): a randomized trial.

[41] Abbas Z. (2015). Gastrointestinal health in Ramadan with special reference to diabetes. *Journal of the Pakistan Medical Association*.

[42] Scheen A. J. (2015). Pharmacodynamics, efficacy and safety of sodium-glucose co-transporter type 2 (SGLT2) inhibitors for the treatment of type 2 diabetes mellitus. *Drugs*.

[43] Kelwade J., Sethi B. K., Vaseem A., Nagesh V. S. (2014). Sodium glucose co transporter 2 inhibitors and Ramadan: another string to the bow. *Indian Journal of Endocrinology and Metabolism*.

[44] http://www.fda.gov/Drugs/DrugSafety/PostmarketDrugSafetyInformationforPatientsandProviders/ucm446852.htm.

[45] Del Prato S., Nauck M., Durán-Garcia S. (2015). Long-term glycaemic response and tolerability of dapagliflozin versus a sulphonylurea as add-on therapy to metformin in patients with type 2 diabetes: 4-year data. *Diabetes, Obesity and Metabolism*.

[46] Kamaruddin N., Seman W. W., Kori N. Assessment of dehydration parameters with dapagliflozin in patients with type 2 diabetes mellitus during Ramadan fasting month.

[47] Leiper J. B., Molla A. M., Molla A. M. (2003). Effects on health of fluid restriction during fasting in Ramadan. *European Journal of Clinical Nutrition*.

